# CircCDR1as upregulates autophagy under hypoxia to promote tumor cell survival via AKT/ERK_½_/mTOR signaling pathways in oral squamous cell carcinomas

**DOI:** 10.1038/s41419-019-1971-9

**Published:** 2019-10-03

**Authors:** Ling Gao, Zhi-Chao Dou, Wen-Hao Ren, Shao-Ming Li, Xiao Liang, Ke-Qian Zhi

**Affiliations:** 1 Department of Oral and Maxillofacial Surgery, the Affiliated Hospital of Qingdao University, 1677 Wutaishan Road, 266555 Qingdao, Shandong China; 2 Key Lab of Oral Clinical Medicine, the Affiliated Hospital of Qingdao University, Qingdao, Shandong China; 3 School of Stomatology, Qingdao University, Qingdao, Shandong China; 4 Department of Neurology, Haukeland University Hospital, 5021 Bergen, Norway

**Keywords:** Oncogenes, Oral cancer, Autophagy

## Abstract

Autophagy, as an important non-selective degradation mechanism, could promote tumor initiation and progression by maintaining cellular homeostasis and the cell metabolism as well as cell viability. CircCDR1as has been shown to function as an oncogene in cancer progression, however, it remains largely unknown as to how autophagy is regulated by circCDR1as in oral squamous cell carcinoma (OSCC). In this study, we validated the functional roles of circCDR1as in regulation of autophagy in OSCC cells and further investigated how circCDR1as contributed to cell survival via up-regulating autophagy under a hypoxic microenvironment by using combination of human tissue model, in vitro cell experiments and in vivo mice model. We found that hypoxia promoted the expression level of circCDR1as in OSCC cells and elevated autophagy. In addition, circCDR1as further increased hypoxia-mediated autophagy by targeting multiple key regulators of autophagy. We revealed that circCDR1as enhanced autophagy in OSCC cells via inhibition of rapamycin (mTOR) activity and upregulation of AKT and ERK_½_ pathways. Overexpression of circCDR1as enhanced OSCC cells viability, endoplasmic reticulum (ER) stress, and inhibited cell apoptosis under a hypoxic microenvironment. Moreover, circCDR1as promoted autophagy in OSCC cells by sponging miR-671-5p. Collectively, these results revealed that high expression of circCDR1as enhanced the viability of OSCC cells under a hypoxic microenvironment by promoting autophagy, suggesting a novel treatment strategy involving circCDR1as and the inhibition of autophagy in OSCC cells.

## Introduction

Oral squamous cell carcinoma (OSCC) is one of the most common malignant tumors worldwide, with over 300,000 cases annually^1,2^. Despite significant progress in radical surgery and chemoradiotherapy has improved the treatment of OSCC, its mortality rate remains basically unchanged (around 48%) and the 5-year survival rate is very poor (<50% overall) in the past few decades^3,4^. Importantly, over 60% of OSCC patients was diagnosed at TNM stage III and IV and exhibited a lower survival rate^5^. As malignant tumors, OSCC was not only composed cancer cells but also composed and surrounded by a complex tumor microenvironment, including hypoxic and nutrient-poor environment as well as chronic inflammation^6^. Tumor microenvironment plays essential roles in tumor initiation and malignant progression, energy metabolism and immune escape^7,8^.

Autophagy is a lysosome-dependent cellular degradation program, which maintains energy metabolism homeostasis by eliminating damaged cellular components that could otherwise become toxic, providing an internal source of nutrient and energy to cells survival in starvation^9^. Autophagy has four key stages including: (a) induction of phase-independent membrane-like structure formation stage; (b) autophagosome formation stage; (c) ubiquitin-like-binding system; and (d) autophagosome maturation degradation stage. Autophagy is activated in response to intrinsic and extrinsic stresses, such as endoplasmic reticulum stress, infection of intracellular pathogens, hypoxic stress, and drug induction, etc., in order to cope with and adapt to the stress and improve cell survival^10^. Recent studies have shown that autophagy plays a critical role in the occurrence of tumors and malignant transformation, neurodegenerative diseases, and inflammatory diseases^11,12^. In advanced stage tumors, cancer cells survive under low-nutrition and hypoxic conditions by inducing autophagy due to cancer cells have higher bioenergy requirements and nutritional needs than normal cells^13^. The elucidation of the association between autophagy and poor survival in various cancers, suggested that autophagy may serve as a marker for both diagnostic and clinicopathological characteristics^14–16^. Thus, understanding the signaling pathways involved in the regulation of autophagy as well as its biological functions in OSCC represents new directions in the development of anticancer therapeutic strategies.

Circular RNA (circRNA) has been identified as a novel member of the noncoding cancer genome, which has distinct properties and diverse cellular functions^17^. Previous studies have demonstrated that overexpression of circCDR1as was associated with an unfavorable prognosis, as well as tumors migration and invasion in various tumors, including colorectal cancer, lung cancer, and hepatocellular carcinoma^18–20^. It was reported that expression of circCDR1as effectively blocked miR-7, resulting in decreasing miR-7 activity and increasing miR-7 targeting transcript levels^21^. However, it is still unclear whether circCDR1as could promote autophagy of OSCC and what is the main role of circCDR1as on triggered autophagy under a hypoxic microenvironment, as well as the underlying mechanisms.

To address these issues, we collected 57 OSCC tissues and their matched tumor-adjacent normal samples to explore the role of autophagy. In addition, commercial OSCC cell lines (Tca-8113 cells and SCC-15 cells) and mice model were further used to detect the mechanism of circCDR1as regulating autophagy. Here, we found that circCDR1as acted as a miRNA-671-5p (miR-671-5p) sponge to promote OSCC cells autophagy. In addition, our study demonstrated that overexpression of circCDR1as inhibited apoptosis in OSCC cells via promoting autophagy under a hypoxia condition, and facilitated the growth of implanted tumors in vitro and autophagy of tumor tissues. Our results were the first to reveal the relationship between circCDR1as and autophagy in OSCC, which may provide a novel strategy for the treatment of OSCC.

## Results

### Hypoxia upregulates autophagy-associated proteins expression levels in OSCC

**Fig. 1:**
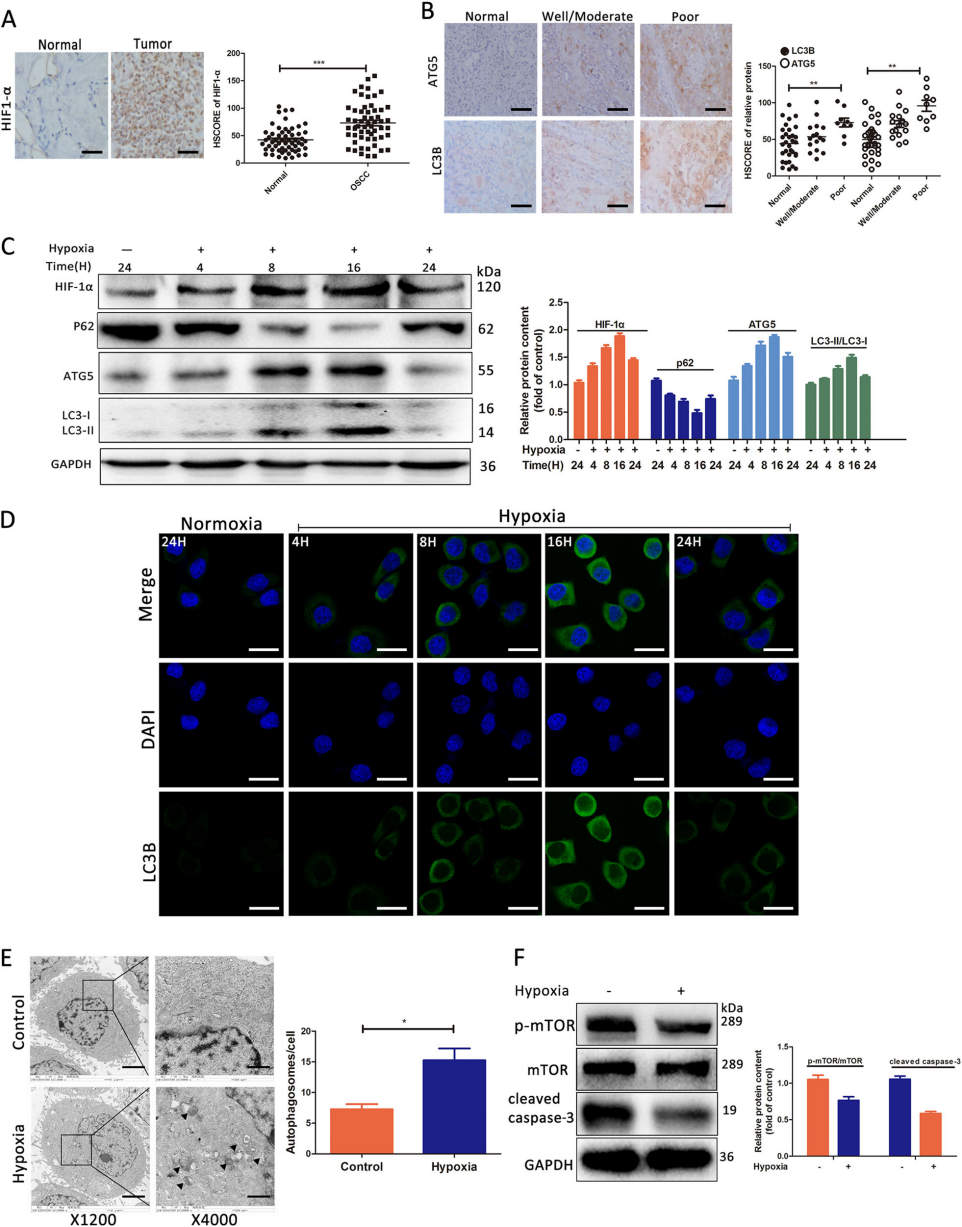
HIF-1α and autophagy-related proteins were upregulated in OSCC tissues, and hypoxia microenvironment triggered autophagy in Tca-8113 cells. **a** Stained tissues are shown at ×400 magnifications, and HSCORE of the two groups. **b** IHC staining for LC3B and ATG5 are shown at ×400 magnifications, and H-SCORE of the three groups. **c** Tca-8113 cells are cultured under a hypoxia condition in different times. The expression levels of LC3 and p62 were tested by western blotting. **d** LC3B distribution detected by immunofluorescence. **e** Tca-8113 cells were treated with or without hypoxia for 16 h and then analyzed by transmission electron microscopy. **f** Tca-8113 cells were treated with or without hypoxia for 16 h and then the expression of p-mTOR, mTOR, and cleaved caspase-3 were tested by western blotting. *p < 0.05

Solid tumors are generally under a hypoxic microenvironment that leads to rapid tumor growth^22,23^. We firstly screened the protein expression of hypoxia-inducible factor-1α (HIF-1α) in a total of 57 OSCC tissues and paired normal tissues. We found that HIF-1α staining was more predominant in the stroma and the epithelial compartments in OSCC tissues compared to that in normal tissues (Fig. 1a). Consistently, the histochemistry scores (HSCORE) of HIF-1α were significantly higher in OSCC samples than in normal samples (*p* < 0.001) (Fig. 1a). Then we performed IHC staining for autophagy-related proteins, LC3B and ATG5 (Fig. 1b). The HSCORE of total LC3B was elevated in OSCC tissues compared to that in normal tissues, and it was related to the degree of tumor differentiation (*p* = 0.004) (Fig. 1b). Similarly, the HSCORE of ATG5 was also upgraded in OSCC samples than in normal samples (*p* < 0.001) (Fig. 1b). To clarify the role of autophagy and hypoxia in OSCC, in vitro culture system with Tca-8113 cells were then conducted and exposed to the different duration of hypoxia (4, 8, 16, and 24 h). We found that the hypoxia had a positive effect on autophagy activation (Fig. 1c–f), a finding consistent with the previous studies^24,25^. After induction of hypoxia exposed to TEM for 16 h, the level of HIF-1α was markedly increased in Tca-8113 cells (Fig. 1c). The level of p62, ATG5, and LC3B (LC3-II) expression was significantly upregulated under hypoxic condition (Fig. 1c, d). Furthermore, autophagic vesicles formation was visualized after 16 h hypoxia (Fig. 1e). In addition, we found that hypoxia downregulated cell apoptosis-related proteins and upregulated autophagy via blocking mTOR pathway (Fig. 1f). These data suggested that hypoxia and hypoxia-associated autophagy were unregulated in OSCC, which might play a role in cell survival in OSCC.

### CircCDR1as further promotes hypoxia-induced autophagy in OSCC cells

**Fig. 2:**
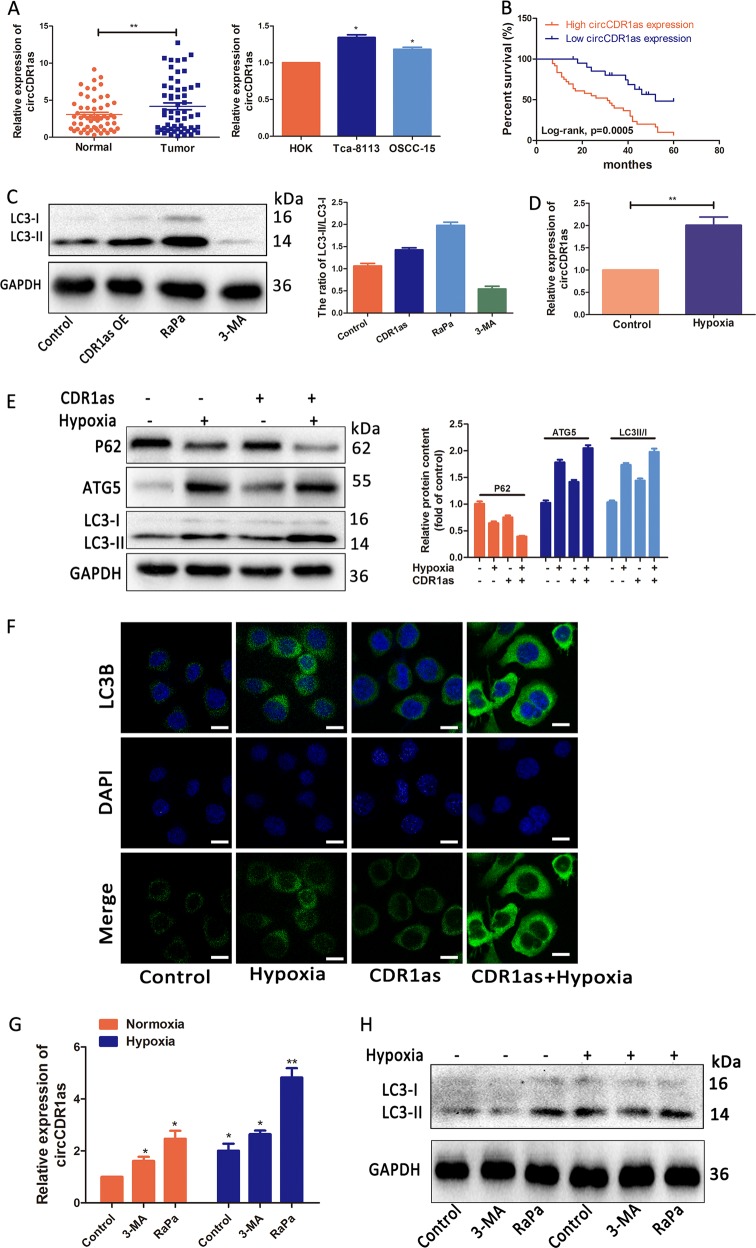
CircCDR1as further promoted autophagy under a hypoxia microenvironment. **a** The circCDR1as expression levels in tissues and OSCC cell lines were determined by qRT-PCR (*n* = 57). **b** The overall survival rate analysis in patients with OSCC using the Kaplan–Meier method. **c** The levels of LC3-I/II in Tca-8113 cells transfected with circCDR1as or treated with 3-MA (5 mM), rapamycin (10 μM), or negative control were examined by western blotting. **d** CircCDR1as levels were enhanced after treated hypoxia in Tca-8113 cells. **e** Tca-8113 cells were transfected with or without circCDR1as, and treated for 16 h with or without hypoxia, then analyzed. **f** Tca-8113 cells were treated as in **c**. Autophagy was analyzed by observation of the immunofluorescence of LC3B (green). **g** Tca-8113 cells treated with autophagy inhibitor and inducer were cultured with or without hypoxia. CircCDR1as levels were enhanced. **h** Tca-8113 cells were treated as in **e**. The levels of LC3I/II were examined by western blotting. *p < 0.05

To explore the significance of circCDR1as in clinical tissues, the expression of circCDR1as in 57 paired OSCC tissues and two OSCC cell lines were conducted by qRT-PCR quantification. As shown in Fig. 2a, circCDR1as expression was strikingly increased in OSCC tissues (*n* = 57, *p* = 0.0034) compared to the normal tissues. Similarly, the expression of circCDR1as in OSCC cell lines Tca-8113 and SCC-15 was up-regulated compared to the human oral keratinocyte cells (HOK) (Fig. 2a). We further investigated the relationship between the circCDR1as expression level and clinicopathological characteristics of OSCC patients (Table 1). Kaplan–Meier analysis demonstrated that patients with a higher circCDR1as expression had shorter postoperative survival time than those with lower circCDR1as expression (ranging from 7 to 60 months, median survival time of 31 months vs. 52 months) (Fig. 2b, *p* < 0.001).

**Table 1:** Relation of circCDR1as expression levels (2^−ΔΔCt^) in cancer tissues with clinicopathological factors of patients with oral squamous cell carcinoma patients

Characteristics	No. of patients (%)	Mean ± SEM	*p*-Value
*Age (year)*			0.586
≤60	22 (38.6%)	3.8445 ± 0.74968	
>60	35 (61.4%)	4.3706 ± 0.59737	
*Gender*			0.146
Male	44 (77.2%)	4.5361 ± 0.55526	
Female	13 (22.8%)	2.5577 ± 0.70938	
*Diameter (cm)*			0.043^*^
≤3	37 (64.9%)	3.5019 ± 0.69599	
>3	20 (35.1%)	5.3990 ± 0.58590	
*Differentiation*			0.133
Well	27 (47.4%)	3.1881 ± 0.62054	
Moderate	21 (36.8%)	4.9852 ± 0.71475	
Poor	9 (15.8%)	5.1978 ± 1.46090	
*Lymphatic metastasis*			0.017^*^
N0	31 (54.4%)	3.1658 ± 0.54822	
N1–3	26 (45.6%)	5.3619 ± 0.72360	
*TNM stage*			0.006^*^
I	8 (14.0%)	2.2088 ± 0.73903	
II & III	31 (54.4%)	3.4800 ± 0.58936	
IV	18 (31.6%)	6.1894 ± 0.83729	

^∗^Indicates statistical significance

Autophagy has been discovered to play a critical role in the tumor occurrence and progression^26^. To further explore the correlation and possible mechanism of interaction of circCDR1as, autophagy and hypoxia, autophagy inducer rapamycin and autophagy inhibitor 3-methyl adenine (3-MA) were treated in OSCC cells. Western blotting analysis showed that the LC3B (LC3-II) expression was elevated in overexpressed circCDR1as cells and cells treated with rapamycin, and decreased in the present of 3-MA (Figs. 2c and S1A). Moreover, the cells with circCDR1as overexpression exhibited enriched LC3 puncta (Fig. S1B), indicating activation of autophagy was induced by circCDR1as. To observe under hypoxic condition, OSCC cells were incubated in a hypoxia chamber. Interestingly, OSCC cells showed a significant increase in terms of the circCDR1as expression after exposure to hypoxic condition (Figs. 2d and S1C). Thus, it has been speculated that circCDR1as, as an oncogene, may undergo biological changes to promote tumor progression under hypoxic condition. These findings promoted us to investigate the possible involvement of hypoxia in promoting the circCDR1as-induced autophagy. The data from western blotting analysis detected the increase ratio of LC3-II/I and ATG5 expression, as well as downregulated p62 levels in circCDR1as overexpressed cells under hypoxic condition compared to those in normoxia culturing conditions (Figs. 2e and S1D). In addition, the images from immunostaining also confirmed that circCDR1as overexpression enable to effectively increased the expression levels of LC3B (Fig. 2f). Therefore, these data suggested that circCDR1as overexpression could enhance cellular autophagy and further promote hypoxia-induced autophagy in OSCC cells.

We then examined whether autophagy activation/inhibition changes the circCDR1as expression profile. Tca-8113 cells were cultured in the presence of 3-MA or rapamycin under normoxic or hypoxic conditions. As shown in Fig. 2g, h, LC3-II/I were downregulated by 3-MA treatment and upregulated by rapamycin; however, the expression of circCDR1as was high with the treatment of rapamycin or 3-MA in these cells. These results suggested that autophagy plays a significant role in the regulation of circCDR1as in OSCC.

### CircCDR1as increases OSCC cells viability through regulation of autophagy and ER stress

**Fig. 3:**
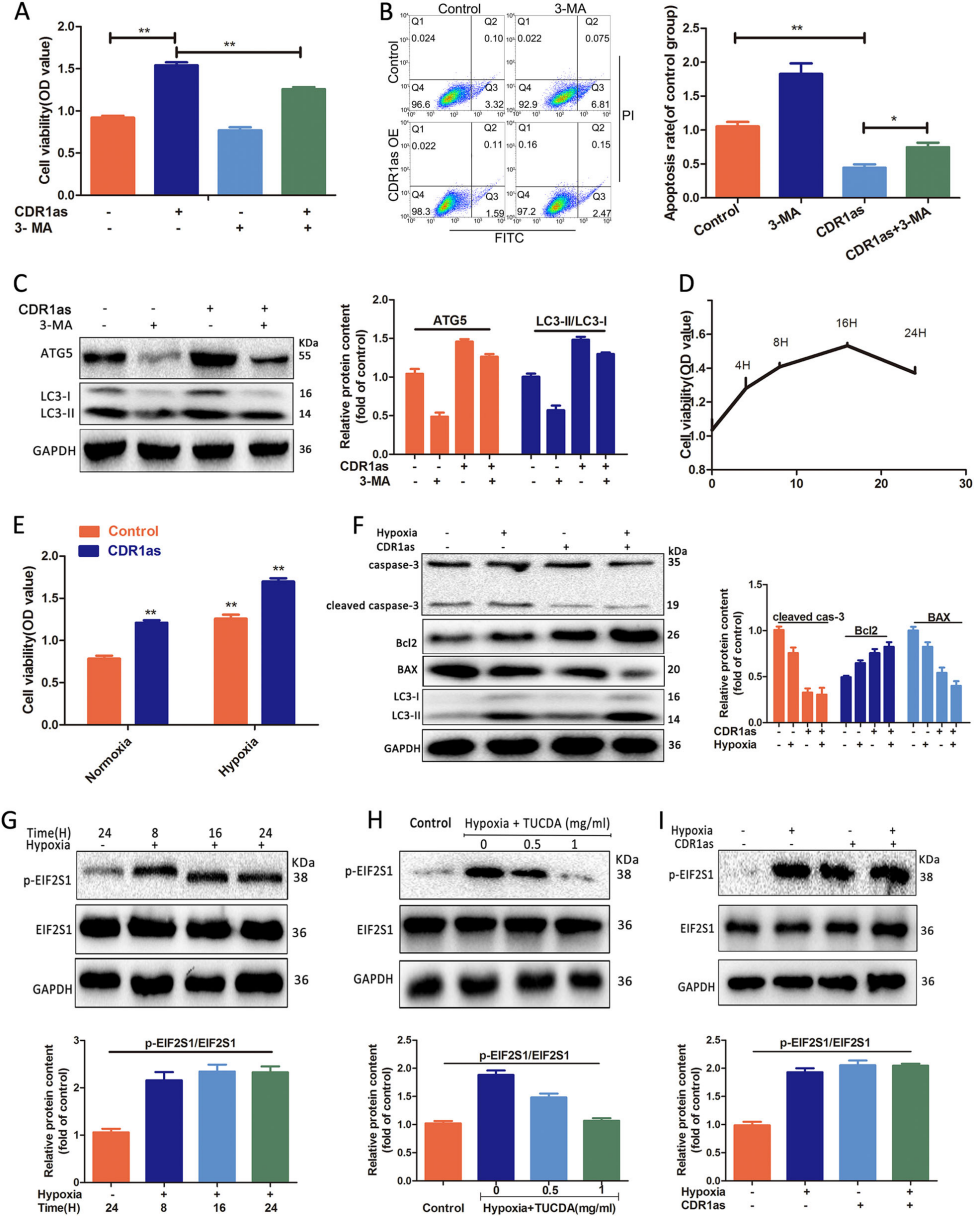
CircCDR1as-induced autophagy further promoted cells proliferation under hypoxia. **a** Suppression of autophagy with 3-MA decreased the viability of transfected circCDR1as cells. **b** Flow cytometry showed that cell apoptosis rate were decreased in overexpression circCDR1as cells, while inhibition of autophagy with 3-MA increased transfected circCDR1as cell apoptosis. **c** Control and circCDR1as overexpression cells were treated with 3-MA or without 3-MA for 12 h. The expression of ATG5 and LC3I/II were measured by western blotting. **d** Tca-8113 cells were treated in a hypoxia microenvironment for different times. The cells viability were measured by CCK-8. **e** Control and circCDR1as overexpression cells were treated with or without hypoxia for 16 h. The cells viability were measured by CCK-8. **f** Figure **f** is grouped in the same way as figure **e**. The expression of caspase-3, cleaved caspase 3, BAX, and Bcl2 were detected by western blotting. **g**, **i** CircCDR1as overexpression and hypoxia effects the ER stress and protein levels were analyzed by western blotting. **h** Cells were treated in a hypoxia microenvironment with the indicated concentrations of TUDCA for 16 h and protein levels were analyzed by western blotting. *p < 0.05

Since the role of autophagy has been extensively studied for its potential functions as “double-edged sword” in cancer cells, including inhibiting the cell survival and tumor development and promoting the tumor metastasis^26^. We then investigated whether circCDR1as-induced autophagy acts as a protective mechanism to promote cell survival in OSCC. CCK-8 analysis showed that the cells treated with autophagy inhibitor 3-MA reduced cell viability induced by the circCDR1as (Figs. 3a and S2A). Furthermore, Fig. 3b exhibited that apoptosis rate was declined after 3-MA treatment in Tca-8113 cells, overexpressed with circCDR1as, suggesting that circCDR1as-induced autophagy promote the growth of Tca-8113 cells. In addition, the ratio of LC3-II/I was increased in overexpression circCDR1as cells in the presence of 3-MA compared to control, suggesting circCDR1as promoted autophagy level (Fig. 3c).

Based on the findings articulated above, we proposed a hypothesis that circCDR1as might contribute to tumor cell survival through upregulating autophagy under hypoxia. In order to confirm our hypothesis, cells were cultured under a hypoxia (2%) condition at different times and cell viability was analyzed by CCK-8 assay. The results showed that cell proliferative activity increased sharply and reached the peak when the duration of hypoxia was 16 h. Then, we performed CCK-8 and western blotting and found that overexpression circCDR1as promoted cell proliferation and further decreased cell apoptosis-related proteins expression (cleaved caspase-3, BAX) after exposure to hypoxia (Figs. 3e, f, and S2B), thereby reflecting that circCDR1as-induced autophagy contributed to OSCC cell survival under a hypoxic condition.

Cells respond to ER stress by misfolding proteins and then cause misfolded proteins accumulation, termed as the unfolded protein response (UPR)^27^. Previous studies have demonstrated that UPR is activated in various cancers and served as a survival strategy for cancer cells^27,28^. Cellular autophagy is affected by ER stress to degrade misfolded proteins. Thus, we further determined whether circCDR1as could activate ER stress in OSCC cells. Firstly, we found that the activiation of ER stress induced by hypoxia, as an external stimulus, was observed at 8 h, and lasts for 24 h via phosphorylating ER stress-related proteins such as eIF2α and p-eIF2α (Fig. 3g). Hypoxic-treated cells showed an attenuated response to ER stress after ER stress had been inhibited by chemical chaperon tuaroursodexycholic (TUCDA) (Fig. 3h). We found that circCDR1as overexpression promoted the ER stress under both nomoxia and hypoxia conditions (Figs. 3i and S2C). Therefore, we inferred that circCDR1as played vital roles in cell survival by regulating ER stress or autophagy in OSCC.

### CircCDR1as enhances autophagy via regulating lysosome activity, AKT/ERK_½_/mTOR signal pathways and ROS under a hypoxic microenvironment

**Fig. 4:**
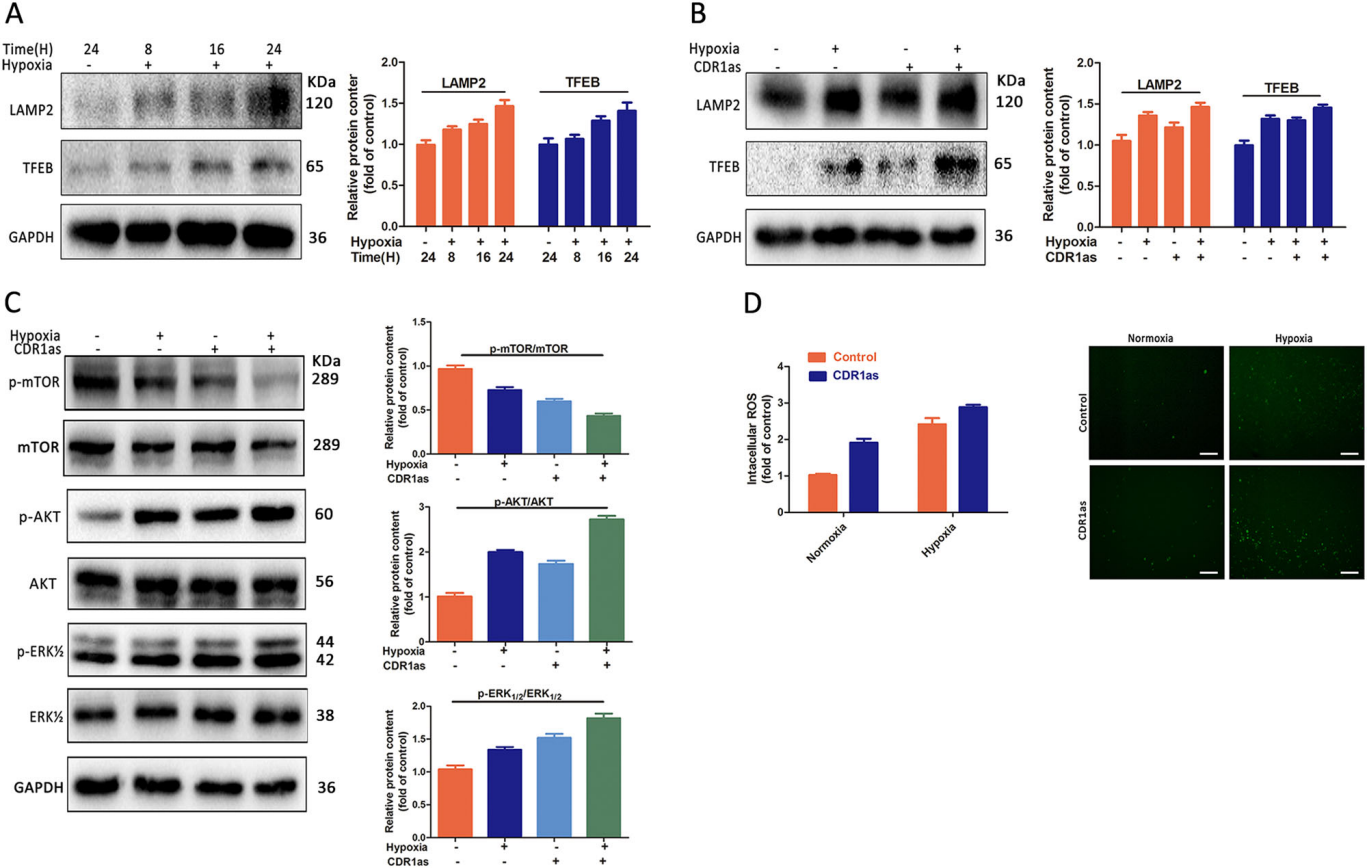
CircCDR1as regulated autophagy of Tca-8113 cells via effecting lysosomal activity and ERK_½_/AKT/mTOR signal pathway. **a**, **b** CircCDR1as overexpression and hypoxia affected the Lysosome-related proteins’ expression. Lysosome-related proteins were assessed by western blotting. **c** Control and circCDR1as overexpression cells were treated with or without hypoxia for 16 h. Western blotting of ERK_½_/AKT/mTOR signal pathway. **d** Figure **d** is grouped in the same way as figure **c**, The ROS were measured using fluorescence microplate reader. *p < 0.05

To initiate autophagy, autophagosomes were formed and subsequently fused with lysosomes to form autophagic lysosomes which lead to degradation of the substance^29^. Thus, we tested whether hypoxia could influence lysosomes to upregulate autophagy. The results demonstrated that the lysosomal-associated membrance protein 2 (LAMP2) levels were steadily increased in Tca-8113 cells treated with hypoxia for 24 h (Fig. 4a). The level of the transcription factor EB (TFEB), as a master regulator for lysosomal and autophagic functions, was also enhanced under hypoxia. In addition, circCDR1as overexpression elevated LAMP2 and TFEB levels in hypoxic cells compared to normoxic cells (Figs. 4b and S3A).

To further investigate the underlying regulation mechanism between circCDR1as and autophagy activation, the ERK_½_/AKT/mTOR signaling pathways were examined based on western blotting analysis. The results showed that hypoxia-activated phosphorylated AKT and ERK_½_ protein, but downregulated the expression of the phosphorylation of mTOR, as a downstream pathway (Fig. 4c). After overexpressed the circCDR1as, this process was further promoted, indicating that circCDR1as could regulate autophagy by activating ERK_½_/AKT/mTOR pathways in OSCC cells.

Then, we examined how ROS levels, considered as an autophagy inducer, were modulated by hypoxia together with circCDR1as^30^. We found that circCDR1as overexpression cells showed higher ROS levels in hypoxic cells compared to normoxic cells (Figs. 4d and S3B), indicating circCDR1as and hypoxia could act as the contributor to cellular autophagy. Collectively, these data suggested that overexpression circCDR1as contributed to the promotion of the hypoxia-induced cytotoxicity via altering lysosomal activity and autophagy.

### CircCDR1as acts as a miR-671-5p sponge to promote autophagy

**Fig. 5:**
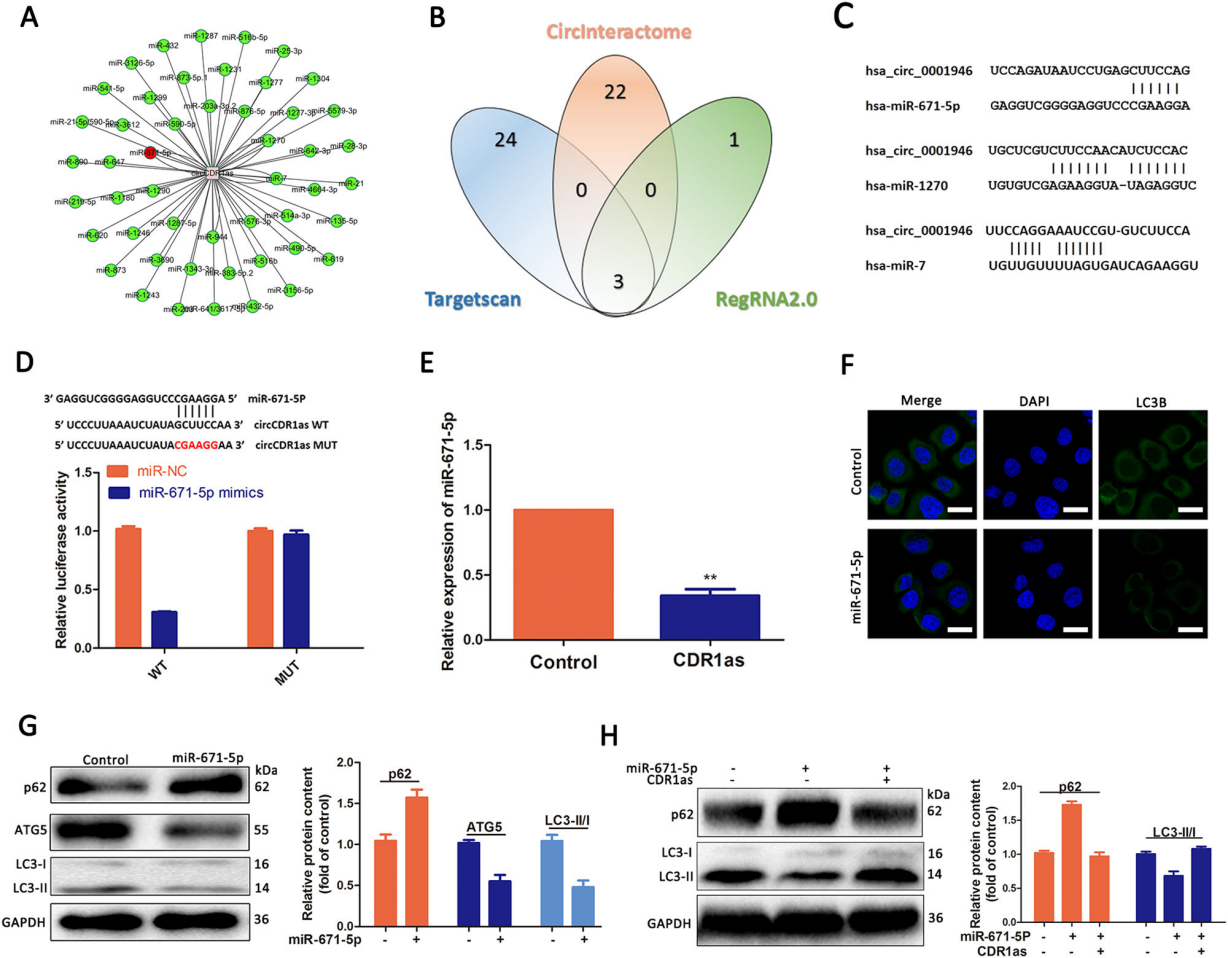
CircCDR1as acted as a sponge for miR-671-5p. **a** The circRNA–miRNA co‐expression network for circCDR1as and miRNAs. **b** Venn diagram of the overlapping parts of the three sets of databases. Three miRNAs in total were common to all databases sets. **c** Three target miRNAs-binding sequence schematic graph. **d** The miR-671-5p-binding site on circCDR1as predicted by targetScan. The association between circCDR1as and miR-671-5p verified by luciferase activity reporter assays. **e** The expression of miR-671-5p in circCDR1as overexpression cells compared with control was detected by qRT-PCR. **f**, **g** Cells were transfected with miR-671-5p mimics or miR-NC, followed by western blot and immunofluorescence to assess autophagy-related proteins. **h** Cells were contransfected with miR-671-5p and circCDR1as. The western blotting analysis was used to examine the protein levels. *p < 0.05

We used TargetScan, CircInteractome (https://circinteractome.nia.nih.gov/), and RegRNA 2.0 database to analyze circCDR1as and a circRNA–miRNA co-expression network was created and analyzed (Fig. 5a). Through the intersection of three databases, we obtained three miRNAs including hsa-miR-671-5p, hsa-miR-7, and hsa-miR-1270. We then analyzed the binding elements and binding sequences schematic diagram of the three target miRNAs on circCDR1as (Fig. 5b). Among these three miRNAs, miR-671-5p was demonstrated to be a possible contributor interacting with circCDR1as (Fig. 5c, d). To further confirm this, we conducted dual-luciferase reporter assay to observe the interaction of circCDR1as and miR-671-5p. Our data demonstrated that the luciferase intensity was attenuated in the cells co-transfected with miR-671-5p and circCDR1as 3′UTR-WT (Fig. 5d). Subsequently, we observed that the expression of miR-671-5p was decreased in circCDR1as overexpressed cells (Fig. 5e). Results above allow us to consider extendedly the possible direct interaction between circCDR1as and miR-671-5p. In order to acquire additional evidence for the role of miR-671-5p on autophagy in OSCC, the Tca-8113 cells transfected with miR-671-5p mimics were used. The declined LC3B(LC3-II) expression level was detected in cells transfected with miR-671-5p mimics compared to that in transfected negative control cells (Fig. 5f, g). Furthermore, promoted autophagy as shown in elevated LC3B(LC3-II) protein expression was identified in OSCC cells overexpressed with circCDR1as and miR-671-5p mimics (Fig. 5h). Thus, these findings confirmed that circCDR1as acted as a sponge for miR-671-5p in OSCC cells.

### CircCDR1as promotes OSCC tumor growth in vivo

**Fig. 6:**
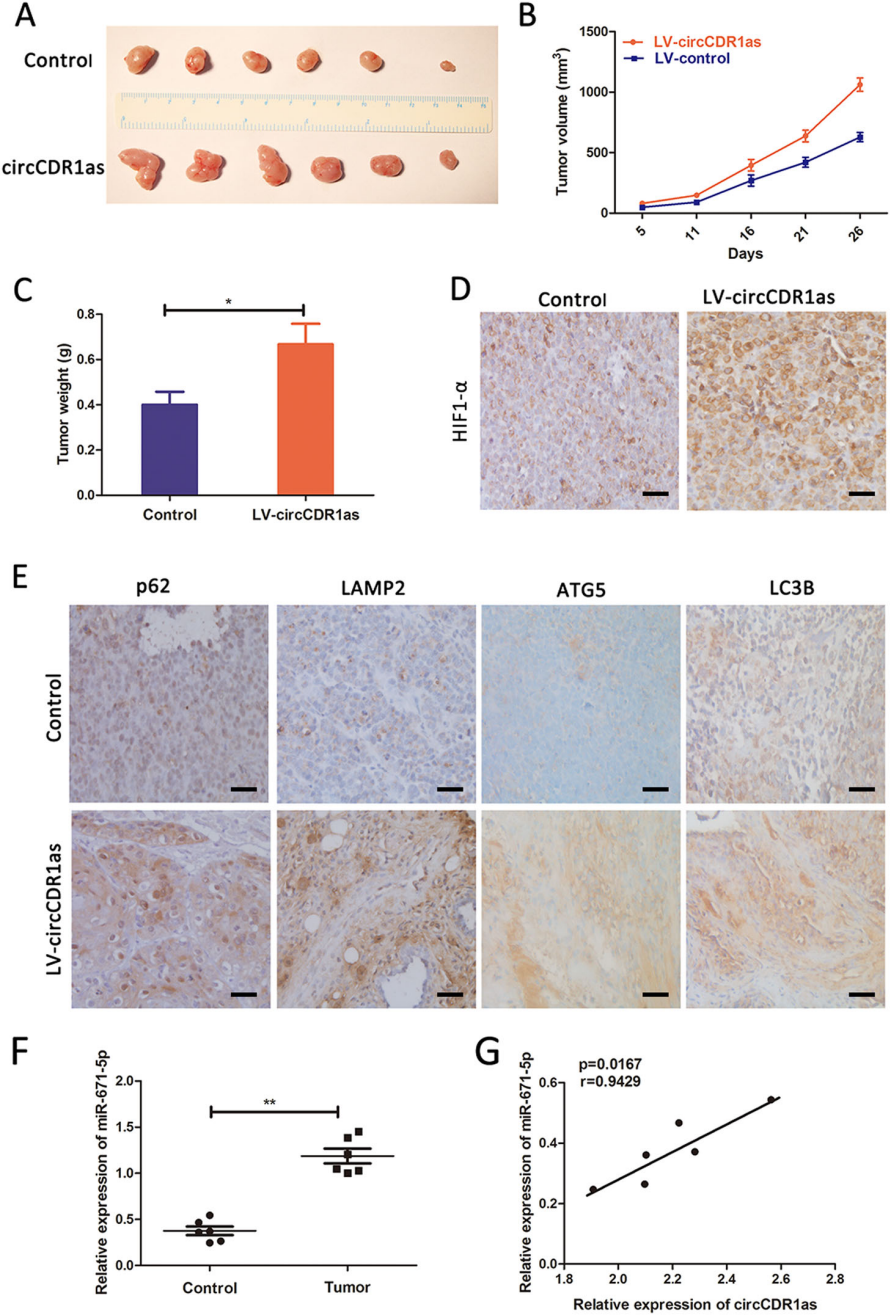
Effect of circCDR1as overexpression on tumorigenesis in vivo. **a**–**c** The tumor size and tumor weight were observed in two groups. **d** The expression of HIF-1α was observed between the two groups. **e** The expression of LC3B, p62 of cytoplasm, LAMP2, and ATG5 were increased in circCDR1as overexpressing group when compared with control group. **f** The expression of miR-671-5p. **g** The correlations of circCDR1as and miR-671-5p were based on the qRT‐PCR results. *p < 0.05

To further verify whether circCDR1as could regulate tumor growth and autophagy in vivo. Tca-8113 cells transfected with circCDR1as lentivirus or control vector were subcutaneously injected into nude mice. As shown in Fig. 6, the results demonstrated that overexpression circCDR1as lentivirus increased the tumor volume and weight on Tca-8113 cell tumor xenograft in nude mice, suggesting that overexpression circCDR1as significantly promoted tumor growth versus the control group. Immunohistochemistry (IHC) staining showed increased autophagy-related proteins expression in the tumor isolated from the mice with injection of circCDR1as cells including LC3B, p62 of cytoplasm, LAMP2, and ATG5, and the expression of HIF-1α were increased in experiment groups compared to the control groups (Fig. 6d, e). In addition, the expression of miR-671-5p was suppressed in overexpressing circCDR1as group compared with control and negatively correlated with the expression level of circCDR1as (Fig. 6f, g). These in vivo results further confirmed the essential roles of circCDR1as in regulation of autophagy, which may partially be the mechanism underlying circCDR1as-mediated OSCC development and progression.

## Discussion

**Fig. 7:**
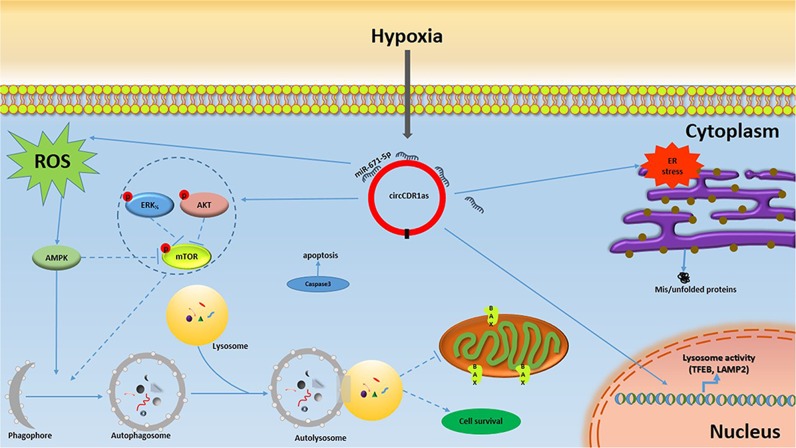
The mechanism underlying the regulation of autophagy

Several studies reported that the overexpression circCDR1as is associated with poor prognosis of human cancer patients^18,19^. Previously, we have investigated that circCDR1as promoted proliferation and invasion through acting as a miR-7 sponge in OSCC (unpublished data). Here, we revealed that overexpression circCDR1as not only promoted OSCC cells proliferation in vitro and the growth of implanted tumors in vivo, but also stimulated cells autophagy. The effects of circCDR1as on cellular autophagy contributed to OSCC progression and development. Particularly under a hypoxia microenvironment, circCDR1as further promoted hypoxia-induced autophagy, which enhanced the OSCC cells survival via modulating AKT/ERK_½_/mTOR/ROS pathways. Furthermore, circCDR1as acted as a miR-671-5p sponge to promote autophagy (Fig. 7).

Autophagy is considered to be a nonselective pathway for the degradation of damaged or unwanted proteins and organelles^9^. In the present study, we first analyzed the clinical function of circCDR1as in OSCC patients (Fig. 2a, b and Table 1) and then confirmed the impact of circCDR1as on induction of autophagy via applying 3-MA and rapamycin treatment in vitro OSCC cells (Figs. 2a, c, 3c, S1A, B). Autophagy is recognized to act as a pro-survival mechanism in cancer cells by eliminating organelles and unfolded/misfolded proteins for nutrient cycling and cellular metabolism^27^. Previous studies revealed that autophagy has “cytotoxic or inhibitory functions” leading to autophagic cell death^31^. However, research implied that autophagy may serve as a double-edged sword depending on the contextual demands placed on tumor cells^32^. Here, we reported that circCDR1as-induced autophagy contributed to resist cell death, indicating it exerted as a protective mechanism for tumor progression. Moreover, depletion of autophagy using the autophagy inhibitor abolished the effect of circCDR1as on OSCC cells survival. In addition, both rapamycin and 3-MA upregulated the circCDR1as expression, suggesting the importance of autophagy on circCDR1as’s effects, and whether autophagy could mediate circCDR1as mutation, degradation, or translocation needed for further investigation. According to our findings, the link between circCDR1as-induced autophagy and OSCC progression was first determined, and autophagy inhibitors combined with circCDR1as could be a new therapeutic target for OSCC.

In our study, IHC staining showed increased LC3B, P62 of cytoplasm level in the tumor isolated from the mice with injection of circCDR1as cells in experiment groups compared to the control groups; while the data from western blotting analysis detected the ratio of LC3II/I was increased, and P62 levels was downregulated in circCDR1as-overexpressed cells under hypoxic condition compared to those in normoxia-culturing conditions. P62 is a multifunctional adapter protein of 440-amino acids that servers as a versatile multitasker during the development of tumors^33^, such as tumor formation and metastasis, regulation of cell cycle and survival, control of oxidative stress response and signal transduction^34,35^. P62 plays different roles in tumor tumorigenesis and metastasis under diverse autophagy conditions. First, P62 has been identified as both an autophagy substrate and adapter protein that links between ubiquitination and autophagy^36^. Defects in autophagy lead to the failure of protein and organelle quality control in cells, which results in the accumulation P62. Recently, P62 has been recognized as a new mediator of oxidative stress signaling in tumorigenesis through the interaction with Kelch-like ECH-associated protein 1 (KEAP1) at the transcription factor NF-E2-related factor 2 (NRF2)-binding site^37^. On the other hand, P62 has different functions in tumor progression in autophagy-defected cells. Previous study showed that P62 accumulation promoted the cell proliferation and migration in the autophagy-defected cells, and promoted tumor growth and metastasis in a mice model^38^. Another study demonstrated that P62 and autophagy synergize promote tumor growth and metastasis^35^. The study on autophagy-deficient established murine tumors showed that increased P62 promoted tumor growth and metastasis via NF-κB signaling and pro-tumorigenic inflammatory cytokines^39^. Therefore, we then speculated P62 might play a dual role in tumor development. The distinct half-life time of LC3B and p62 might be also the other possible explanation for this paradoxical phenomenon.

As known, hypoxia occurs after solid tumor occurrence and is induced by the extreme energy demands of rapidly dividing cells. Although hypoxia can lead to apoptosis or necrosis, it can also prevent cell death by stimulating cells to produce some adaptive responses as a protection mechanism contributing to cancer progression^40^. In the present study, we confirmed that cellular autophagy and ER stress-mediated UPR were stimulated in responses to hypoxia via observing autophagy and ER stress-related proteins in OSCC cells. In addition, we found that the expression of LC3B/ATG5 were associated with the different differentiation stages of OSCC, suggesting treatment together with autophagy inhibitors may be valuable for the management of advanced OSCC. More importantly, we investigated that hypoxia-mediated apoptosis was decreased in OSCC cells, where autophagy was promoted and confirmed that autophagy was induced via blocking mTOR activation. These data confirmed the hypothesis that autophagy induced by hypoxia may serve as a survival strategy for OSCC cells. Previous studies found that HIF-1α was associated with the development and poor prognosis in many cancers, including gastric cancer, breast cancer, and lung cancer, and could change the biological function of a series of genes^41–43^. Interestingly, circCDR1as expression was increased by hypoxia exposure and played a role in tumor growth under a hypoxic environment in OSCC. However, the role of HIF-1α pathway in cellular adaptation to hypoxic and the upregulation of the circCD1as expression as well as the HIF-1α-independent regulation mechanism of circCDR1as expression need to be further explored. Furthermore, circCDR1as further promoted hypoxia-induced autophagy and UPR to further effect on development of xenograft tumor and OSCC cells viability. Therefore, these results supported that circCDR1as acted as an oncogenic biomarker to promote OSCC progression, and indicated that HIF-1α, LC3B, and p62 expression were molecular markers of OSCC because they were found in OSCC tissues.

Several signaling pathways regulate autophagy. Previous studies have fully confirmed the signaling pathways involved in autophagy, including mTOR, PI3K-AKT, and MAPK/ERK_½_; and mTOR is a key regulator of autophagy induction^44–46^. To determine the downstream mechanisms by the effect of circCDR1as on autophagy under a hypoxia microenvironment, the potential involvement of the mTOR-signaling pathway was assessed. In this study, we found that circCDR1as further promoted the hypoxia-induced activation of AKT and ERK_½_ and inhibition of mTOR activity. Furthermore, ROS accumulation was induced by circCDR1as upregulation and considered to act as a positive role in autophagy activation under a hypoxic environment in OSCC. This is the same as shown in the previous reports where ROS has been identified as a cellular autophagy regulator by inhibiting mTOR pathway activation^47^. Indeed, ROS also plays a key role in the transduction pathways and promotes cancer cell survival and metastasis in malignant cells^48,49^. Therefore, it would be necessary to determine the circCDR1as-induced ROS for the OSCC survival and metastasis under hypoxic conditions and their underlying mechanism of circCDR1as-induced autophagy in the future studies. Notably, TFEB coordinates autophagosome degradation by driving expression of autophagy and lysosomal genes^50^. Our data showed that circCDR1as induced increased levels of TFEB and LAMP2, which is an essential regulator for lysosomal function. Both hypoxia and circCDR1as not only promoted the formation of autophagy vesicles but also promoted the fusion of lysosomes and autophagosomes via regulating TFEB and LAMP2. These results highlighted that AKT/ERK½/mTOR/ROS pathways and lysosomal activity contributes to circCDR1as-induced autophagy activation in hypoxic conditions in OSCC, which might offer new and potentially important targets for the therapy of OSCC.

CircRNAs, forming a circular structure via splicing the 3′ end and the 5′ end, were characterized with highly stable and were largely resistant to degradation^51^. In the past several decades, circRNAs were found to sponge microRNAs (miRNAs) and then suppress their functions, circRNA sponge activity will affect miRNAs’ biological behaviors^52,53^. In our other research, we have revealed that overexpression circCDR1as could attenuate anti-metastatic effects of miR-7 on OSCC cells, suggesting that miR-7 is interacting with circCDR1as in OSCC (unpublished data). In this study, we predicted putative-binding sites between circCDR1as and miR-671-5p based on bioinformatics analysis. Then, the relationship between circCDR1as and miR-671-5p was identified in cytoplasm through dual-luciferase reporter assay and qRT-PCR. Our findings demonstrated that circCDR1as regulated autophagy by sponging miR-671-5p in OSCC cells.

In summary, our results demonstrated that circCDR1as could trigger activation of autophagy to protect OSCC cells from apoptosis, and circCDR1as-induced autophagy adapt to the distressed hypoxic microenvironment. Thus, inhibition of autophagy in combination with circCDR1as may be a potential therapeutic strategy.

## Materials and methods

### Patients and specimens

A total of 57 matched pairs of OSCC and adjacent normal tissues were collected from patients who underwent surgeries at the Department of Oral and Maxillofacial Surgery, the Affiliated Hospital of Qingdao University, between June 2014 and December 2018. No patients received chemotherapy or radiotherapy prior to surgery and intraoperative frozen pathology shows negative margins at the primary lesion. OSCC tissues and paired adjacent normal tissues were obtained, and no tumor cells were found in the paired adjacent tissues. All sample profiles were confirmed by histological examination. This study was approved by the ethics committee of the Affiliated Hospital of Qingdao University. All patients signed an informed consent form.

### Cell culture, hypoxia treatment, and transfection

Cell lines Tca-8113, SCC-15, and HOK were purchased from the Cell Bank of Type Culture Collection of the Chinese Academy of Sciences (Shanghai, China). The cells that were cultured in RPMI-1640 (RPMI 1640, Gibco, USA) or Dulbecco’s modified Eagle’s medium (HyClone, USA) contained 10% fetal bovine serum (FBS) and 1% penicillin–streptomycin at 37 °C in a humidified 5% CO_2_ atmosphere. Hypoxia treatment was performed using a tri‑gas incubator (Thermo, MA, USA) consisting of 5% CO_2_, 93% N_2_, and 2% O_2_ for different periods (4, 8, 16, and 24 h).

MiR-671-5p mimics or Negative Control were designed and synthesized by GenePharma. The sequence of miR-671-5p mimics was 5′-AGGAAGCCCUGGAGGGGCUGGAG-3′. Cells were transfected with the oligonucleotides using Lipofectamine 3000 (Invitrogen, USA) following the manufacturer’s instructions. CircCDR1as-coding sequence was cloned into pLCDH-ciR vector (Geenseed Biotech, Guangzhou, China). Then, circCDR1as stable overexpression cell lines were constructed following the manufacturer’s instructions. The efficiency of circCDR1as overexpression was confirmed by qRT-qPCR.

### Reagents and antibodies

All reagents were purchased either from Thermo Fisher Scientific (Waltham, MA, USA) or Sigma-Aldrich (St. Louis, MO). The antibodies used in the research were as follows: HIF-1α (#36169, CST), p62/SQSTM1 (#23214, CST), BAX (#14796, CST), Bcl2 (#15071, CST), caspase-3 (#9662, CST), LC3-I/II (#12741, CST), ATG5 (#12994, CST), eIF2a (#5324, CST), p-eIF2a (#3398, CST), LAMP2 (#49067, CST), TFEB(#37785, CST), mTOR (#2983, CST), p-mTOR (#5536, CST), and GAPDH (#5174, CST), p-ERK_½_ ((#4376, CST), ERK_½_ (16443-1-AP, Proteintech), AKT (10176-2-AP, Proteintech), p-AKT (66444-1-Ig, Proteintech).

### Quantitative real-time PCR analysis

Total RNA was extracted using RNA reagent Trizol (Thermo Fisher Scientific). The Bio-Rad CFX96 PCR machine was used for the quantitative real-time PCR (qRT-PCR) using PrimeScript™ RT Reagent Kit (Takara, Dalian, China). All primers used for qRT-PCR were listed in Table 2. GAPDH and U6 were used to normalize target gene transcript levels. The values were expressed using the 2^−(ΔΔCt)^ method.

**Table 2:** Primer sequences

Primer set	Forward primer	Reverse primer
circCDR1as	CGGGTCTTCCAGGAAATCCG	TCCGGAAGATGTGGATTGACTG
miR-671-5p	AGTTGTTGGAAGACCTTGACAC	ACCTAACACTAAGGATCACGGA
GAPDH	TCAAGGCTGAGAACGGGAAG	TCGCCCCACTTGATTTTGGA
U6	ACGAATACCGGCGTGAGAAA	TCGTGAAAGACCGCAGCAAA
miR-671-5p RT	GTCGTATCCACTCCAGGGTCCGAGGTATTCGCACTGGATACGACCTCCAG	

### Western blotting analysis

Total proteins were extracted by using the kit based on manufacturer’s instructions. We measured the protein concentration by using a BCA kit (Pierce, USA). Protein samples were loaded onto SDS-containing polyacrylamide gels, separated via electrophoresis, and transferred the separated protein samples onto a polyvinylidene fluoride (PVDF) membrane (Millipore, Billerica, MA, USA). After blocking with 5% BSA in TBST buffer for 2 h, the membrane was incubated at 4 °C overnight with the primary antibody mentioned previously. The membranes were then incubated with secondary antibodies conjugated with horseradish peroxidase for 2 h at room temperature. Finally, images were acquired using ChemiDoc Touch Imaging System (BioRad). Quantification of bands was by densitometry using ImageJ software (National Institutes of Health).

### Immunohistochemical (IHC) analysis

The paraffin-embedded tumor tissue sections were dewaxed, rehydrated, and permeabilized with 0.2% Triton X-100. Slides were incubated overnight at 4 °C with primary antibodies. Then, we used phosphate buffer saline (PBS) to wash them twice and used secondary antibody to incubate tissues at room temperature. Subsequently, we used 3,3-diaminobenzidine solution and hematoxylin to stain the samples and mounting under a coverslip. The images were observed under an Olympus CX31 microscope (Olympus, Tokyo, Japan). Histochemistry scores (Hscores) were calculated as a function of the percentage of positive cells multiplied by the staining intensity (ranging from 0 to 4).

### Immunofluorescence (IF) analysis

For IF assays, cells were cultured on glass coverslips. After different treatments, cells were washed with PBS and fixed with 4% PFA. The cells were permeabilized with 0.2% Triton X-100 followed by blocking in 1% BSA. Primary antibodies were incubated overnight at 4 °C. The cells were washed again for three times in PBS and then incubated with fluorochrome-conjugated second antibody for 1 h at 37 °C. For further nuclear staining, cells were incubated with 4′,6′-diamidino-2-phenylindole (DAPI). Finally, the fluorescence-labeled cells were mounted with ProLong antifade reagent (Invitrogen) and photographed using a Leica SP5 confocal fluorescence microscope (Leica, Wetzlar, Germany).

### Cell viability assay

Cell viability was measured using the cell proliferation assay. Cells were seeded in 96-well plate and incubated overnight in complete medium. Cell survival was evaluated using the Cell Counting Kit (CCK)-8 (Dojindo Laboratories, Tokyo, Japan) according to the manufacturer’s instructions. The absorption at 450 nm was measured with a microplate spectrophotometer (Molecular Devices, Sunnyvale, CA, USA).

### Apoptosis analysis

Cells after treatment were harvested and resuspended in binding buffer at a final concentration of 1 × 10^6^ cells/ml. Briefly, 5 × 10^5^ cells were stained with Annexin V-FITC (5 μl) 100 µg/ml PI (1 µl) in 100 µl binding buffer and incubated for 15 min at room temperature in dark. The samples were analyzed by flow cytometer (Beckman Coulter, Palo Alto, CA, USA).

### Transmission electron microscopy (TEM)

Standard TEM was performed to monitor the ultrastructure of the cells. The Tca-8113 OSCC cells were fixed with 2.5% glutaraldehyde and 1% OsO_4_. Thereafter, ethanol and propylene oxide were applied to dehydrate the samples. Thin slide sections were cut and stained with 0.3% lead citrate. The autophagosomes were detected by using a JEM-1010 transmission electron microscope (JEOL, Tokyo, Japan).

### Detection of intracellular ROS

Tca-8113 cells were seeded in black 96-well plate. After treatment, cells were incubated with 10 μM dichloro-dihydro-fluorescein diacetate (DCFH-DA) probe at 37 °C for 20 min. After incubation, cells were immediately submitted to a fluorescence microscope (Olympus, Tokyo, Japan) and a fluorescence microplate reader. The fluorescence was recorded at excitation/emission of 488/525 nm.

### Luciferase reporter assay

Cells were co-transfected with wild-type (WT) or mutated (MUT) circCDR1as 3′UTR reporter plasmids, and with miR-671-5p mimics or negative control using Lipofectamine 3000 according to the manufacturer’s protocol. At 48 h after transfection, firefly and Renilla luciferase activities were measured consecutively by using a Dual-Luciferase Reporter Assay System (Promega, USA).

### In vivo mice model

Animal experiments were approved and conducted in accordance with the institutional guidelines for animal of the Animal Care and Use Committee of the Affiliated Hospital of Qingdao University. Five-week-old female BALB/c nude mice were supplied by Sino-British Sipper/BK Lab Animal Ltd. (Shanghai China) and were placed in a specific-pathogen-free at the Department of Laboratory Animals. Then, lv-sh_NC or lv-sh_circCDR1as Tca-8113 cells (5 × 10^6^) in 100 μl PBS were subcutaneously injected bilaterally into mice. The tumor volume was measured every 4 days and the tumor volume were calculated as *V* = *a* × *b*^2^ × 0.5. When the average tumor volume reached 100 mm^3^, tumors were removed and the tumors’ weights were measured.

### Statistical analysis

All statistical tests were performed with GraphPad Prism 5.0 (GraphPad Software, La Jolla, CA) and SPSS 18.0 (IBM, Chicago, IL). One-way ANOVA, Paired *t*-test and independent *t*-test were used in this study correctly. Data were expressed as mean ± SEM. Statistical significance was defined as *p < 0.05, **p < 0.01, ***p < 0.001.

## Supplementary information

Figure legends

Figure S1

Figure S2

Figure S3
