# Supplementary material for: CircCDR1as upregulates autophagy under hypoxia to promote tumor cell survival via AKT/ERK½/mTOR signaling pathways in oral squamous cell carcinomas
Source: Cell Death Dis. 2019 Oct 3;10(10):745. doi: 10.1038/s41419-019-1971-9 (PMC6776509; doi:10.1038/s41419-019-1971-9)
Supplement: Supplementary file 1 — Figure legends [file 41419_2019_1971_MOESM1_ESM.docx]

**Figure legends**

**Figure S1:** CircCDR1as further promotes hypoxia-induced autophagy in SCC-15 cells. **A-B,** SCC-15 cells were transfected with or without circCDR1a. Autophagy was analyzed by western blot and immunofluorescence of LC3B (green). **C,** CircCDR1as levels were enhanced after treated hypoxia in SCC-15 cells. **D,** SCC-15 cells were transfected with or without circCDR1as, and treated with or without hypoxia, then analyzed. *P < 0.05.

**Figure S2:** CircCDR1as-induced autophagy further promoted cells viability and ER stress in SCC-15 cells under hypoxia. **A,** Suppression of autophagy with 3-MA (5 mM) decreased the viability of SCC-15 cells overexpressing circCDR1as. **B-C,** Control and overexpression circCDR1as cells were treated with or without hypoxia. The cells viability were measured by CCK-8 and ER stress-related protein levels were analyzed by western blotting. *P < 0.05.

**Figure S3:** CircCDR1as regulates autophagy via effecting lysosomal activity and ERK_½_/AKT/mTOR/ROS pathway in SCC-15 cells under hypoxia. **A,** Control and overexpression circCDR1as cells were treated with or without hypoxia. Lysosome-related proteins was assessed by western blotting. **B,** Figure B is grouped in the same way as Figure A, The ROS were measured using fluorescence microplate reader. *P < 0.05.
