# Supplementary figures and images for: CircCDR1as upregulates autophagy under hypoxia to promote tumor cell survival via AKT/ERK½/mTOR signaling pathways in oral squamous cell carcinomas
Source: Cell Death Dis. 2019 Oct 3;10(10):745. doi: 10.1038/s41419-019-1971-9 (PMC6776509; doi:10.1038/s41419-019-1971-9)

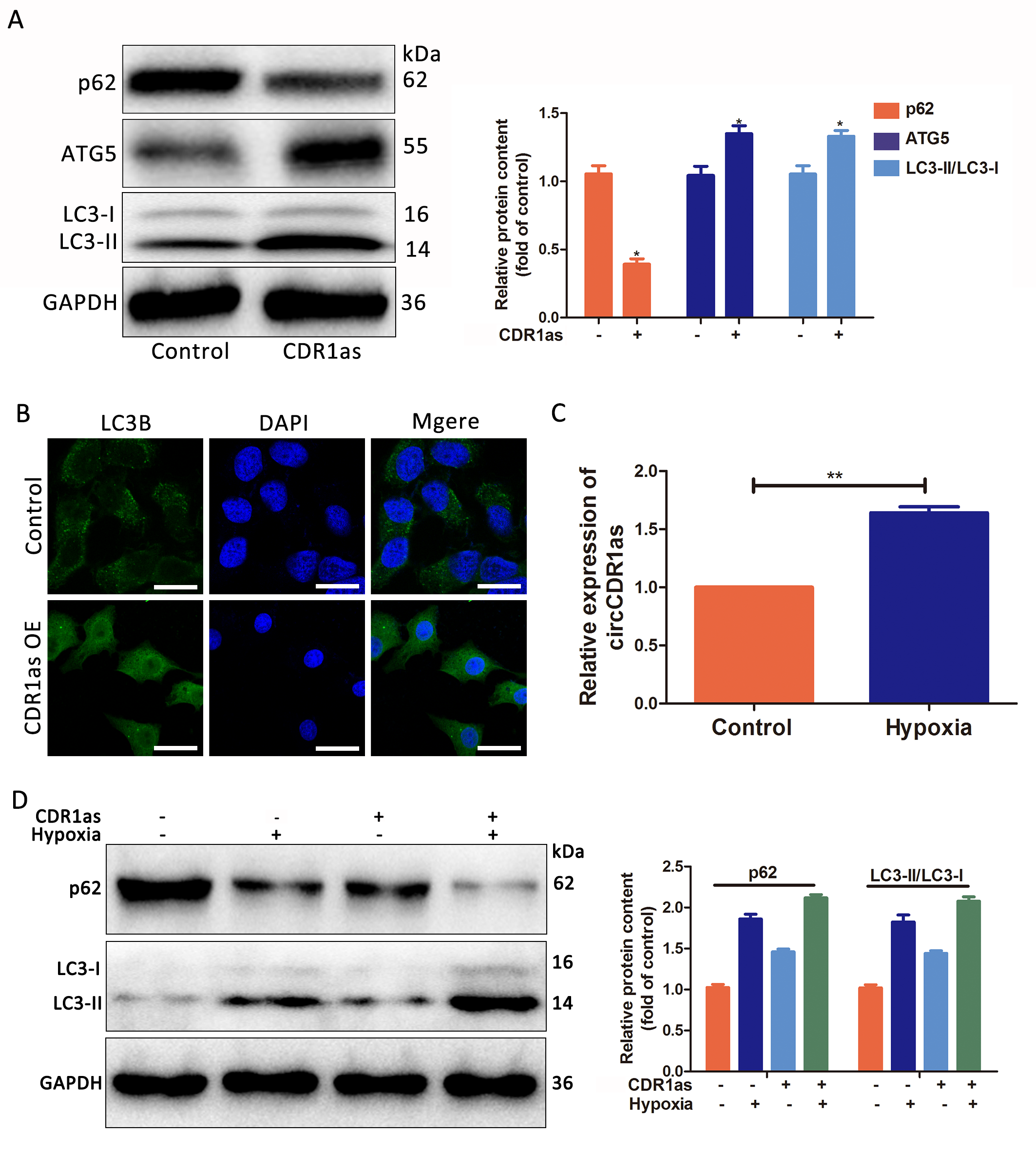

Supplement: Supplementary file 2 — Figure S1 [file 41419_2019_1971_MOESM2_ESM.tif]

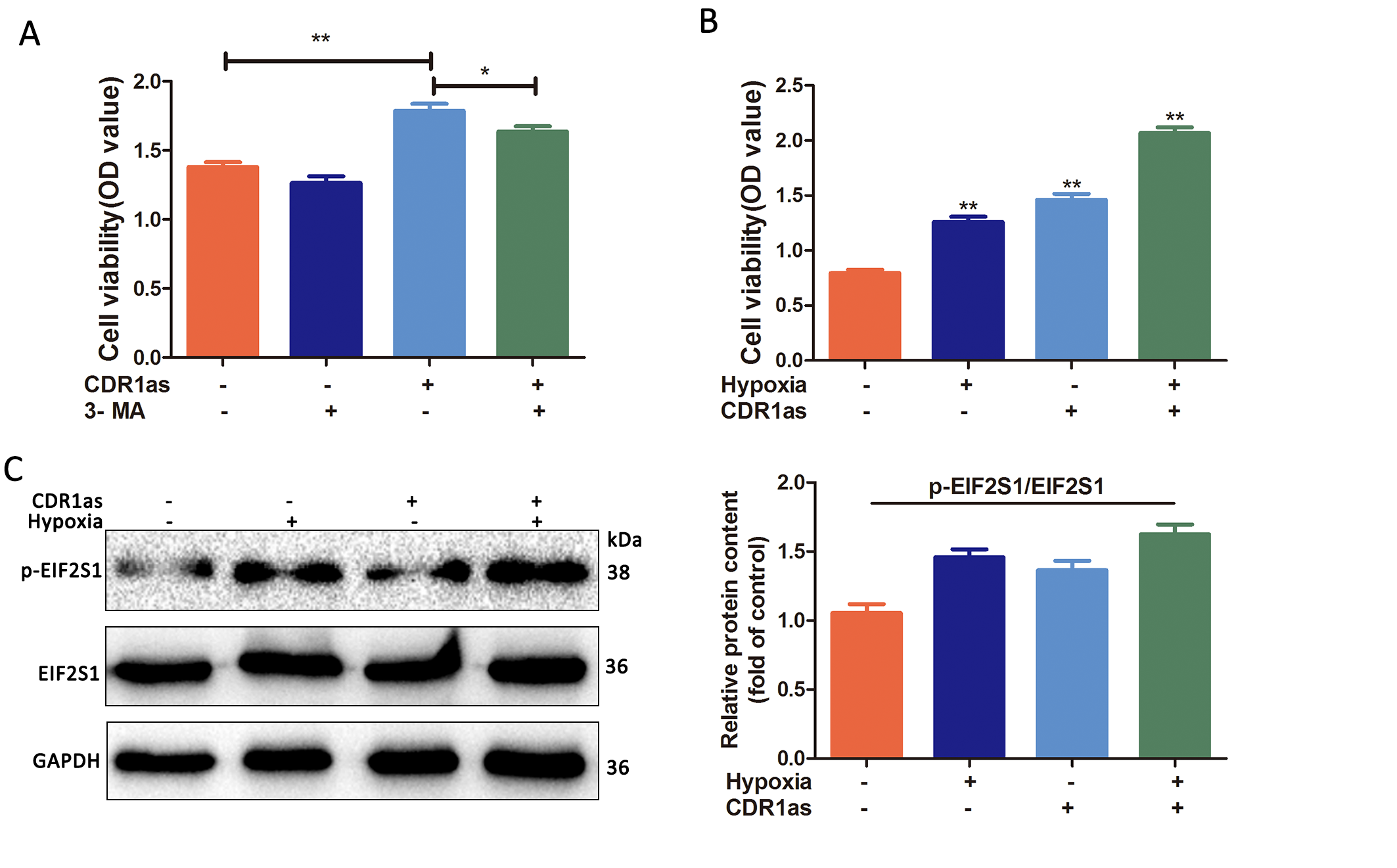

Supplement: Supplementary file 3 — Figure S2 [file 41419_2019_1971_MOESM3_ESM.tif]

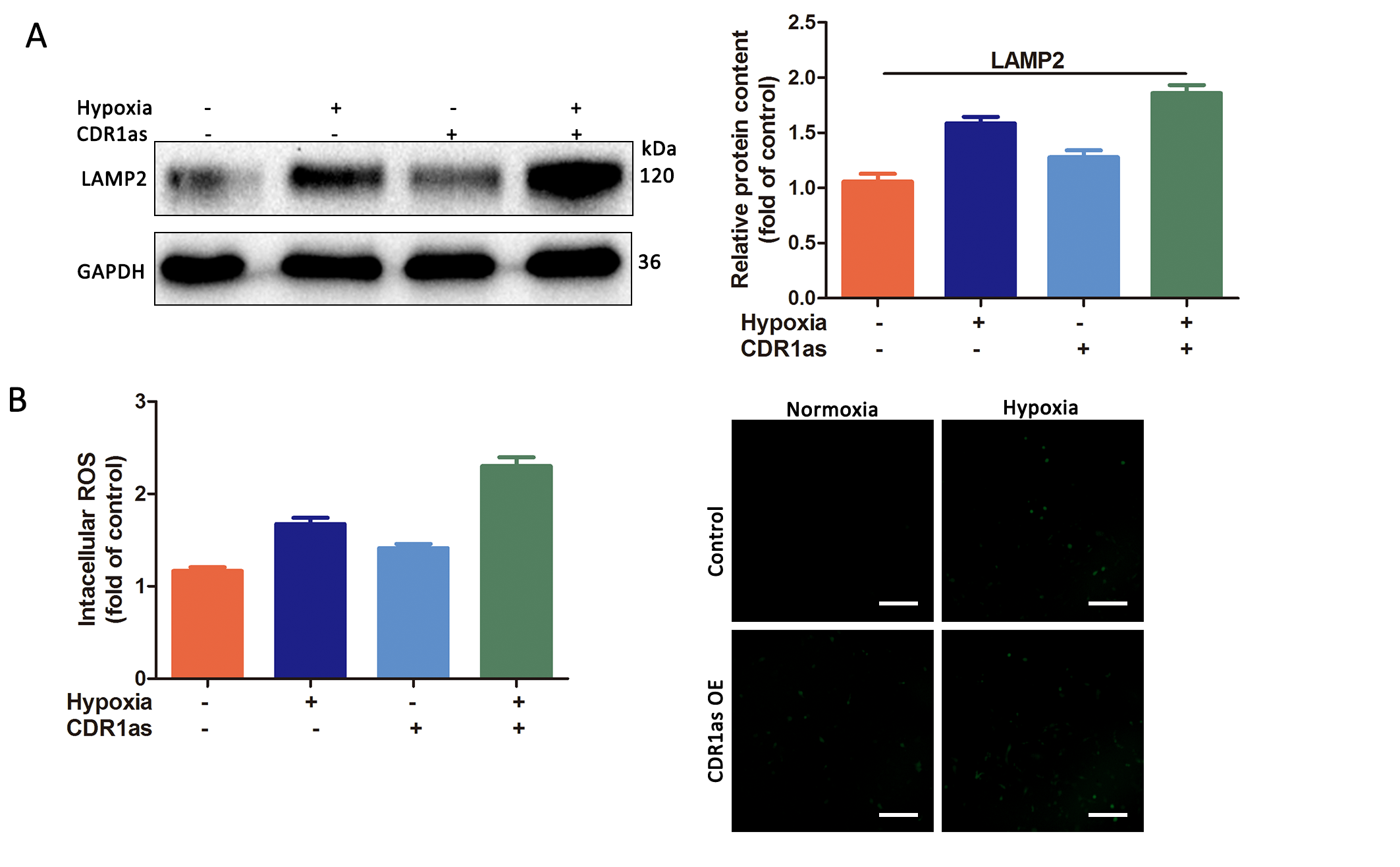

Supplement: Supplementary file 4 — Figure S3 [file 41419_2019_1971_MOESM4_ESM.tif]
